# Populations of the Beet Cyst Nematode *Heterodera schachtii* Exhibit Strong Differences in Their Life-History Traits Across Changing Thermal Conditions

**DOI:** 10.3389/fmicb.2018.02801

**Published:** 2018-11-16

**Authors:** Sylvain Fournet, Lucile Pellan, Catherine Porte, Christophe Piriou, Eric Grenier, Josselin Montarry

**Affiliations:** IGEPP, INRA, Agrocampus Ouest, Université de Rennes 1, Le Rheu, France

**Keywords:** adaptation, genetic diversity, hatching, specialist/generalist, survival

## Abstract

It is widely accepted that climate has an essential influence on the distribution of species and that temperature is the major abiotic factor that affects their life-history traits. Species with very restricted active dispersal abilities and a wide geographical distribution are thus expected to encompass distinct populations adapted to contrasted local conditions. The beet cyst nematode *Heterodera schachtii* is a good biological model to study temperature adaptation in populations collected from different environments. Here, we tested the effect of temperature on *H. schachtii* life-history traits using seven field populations from Morocco, Spain, France, Germany, Austria, Poland and Ukraine. We tested hatching and multiplication rates of each population at different temperatures, as well as hatching rates of each population after storage at different temperatures – simulating survival conditions during the inter-cropping period. Results showed a strong temperature effect on the life-history traits explored. Temperature impact on hatching (at different temperatures and after storage at different temperatures) depended on the origin of populations, separating southern from northern ones. Surprisingly, low temperatures influenced hatching less in southern populations. However, for these populations, a storage period at low temperatures strongly reduce subsequent hatching. Conversely, nematode multiplication was not differentially affected by temperatures, as favorable conditions for the host are also favorable for the parasite. Finally, a significant correlation between the genetic diversity and the level of specialization showed that the less diverse populations were more specialized than the more diverse ones.

## Introduction

There is no doubt that climate has a major influence on the natural distribution of species (Pearson and Dawson, 2003) and that temperature is the major abiotic factor that affects species phenology (Scranton and Amarasekare, 2017). In a context of ongoing climate change, predicting the temperature impact on life-history traits, such as fecundity, development and survival, and thus on the distribution of species is a very challenging scientific question with implications for conservation biology, ecology, agronomy and pest management.

Species characterized by restricted active dispersal ability and a wide geographical distribution are expected to comprise distinct populations adapted to contrasted local conditions (Davis and Shaw, 2001). Plant pathogens, being often distributed across a wide range of climates, are good biological models to study the temperature adaptation of populations from different environments. For instance, patterns of local adaptation to temperature have been highlighted in natural (Laine, 2008) and agricultural host-pathogen systems (Zhan and McDonald, 2011; Mboup et al., 2012; Mariette et al., 2016).

The life cycle of many plant pathogens can be split into two alternating phases: the epidemic period, onto or into the host, and the inter-epidemic period, often outside the host. Several investigations have shown the necessity to take into account the inter-epidemic period in order to better describe the evolutionary trajectories of plant pathogens (e.g., Pfender and Vollmer, 1999; Abang et al., 2006; Montarry et al., 2007; Sommerhalder et al., 2011; Suffert et al., 2015; Pasco et al., 2016). Interestingly, temperature regimes are likely to differ across locations much more during the inter-epidemic periods than during the epidemic periods, because optimal temperatures for host growth is quite similar whatever the geographical locality.

The beet cyst nematode *Heterodera schachtii*, which is regarded as the most important pest in sugar beet production worldwide (Müller, 1999; Amiri et al., 2002), is globally distributed with populations living in highly contrasted environments (see Subbotin et al., 2010 for a review). In European wild beets, *H. schachtii* is relatively rare in south of Spain and Portugal, but more frequent from the northern coast of Spain to Denmark (Gracianne et al., 2014). In European sugar beet fields, *H. schachtii* is widely present from east to west, from Portugal to Ukraine, and from south to north, from Morocco to Finland (Subbotin et al., 2010). Also, as for all plant parasitic nematodes, *H. schachtii* has only very limited active dispersal capabilities in the soil (Wallace, 1968), and temperature is one of the most influential environmental factor affecting key steps of its biological cycle (Trudgill et al., 2005; Kakaire et al., 2012; Kaczmarek et al., 2014; Vandenbossche et al., 2015). Indeed, temperature can act on all or part of life-history traits involved in embryogenesis, hatching and development after infection and survival (Wallace, 1955; Thomason and Fife, 1962; Jones, 1975; Griffin, 1988). For *H. schachtii*, such influence is well known and extensively described in many previous studies, but on a limited number of field populations coming from north European fields (e.g., Vandenbossche et al., 2015). Therefore, *H. schachtii* populations may be thus assumed to be differentially adapted to local temperatures, both for traits involved in development within their host plant (sugar beet), and for traits involved in their survival in the soil when plants are not present.

Here, we explored the effect of temperature on *H. schachtii* life-history traits using seven field populations from Morocco, Spain, France, Germany, Austria, Poland, and Ukraine. We tested the hatching and the multiplication rates of each population at different temperatures, as well as the hatching rate of each population after storage at different temperatures, simulating survival conditions during the inter-cropping period. Finally, to test the hypothesis that greater genetic diversity is associated with less ecological specialization, we calculated the level of specialization to the temperature and assessed the genetic diversity using microsatellite markers for each population.

## Materials and Methods

### Nematode Populations

The seven *H. schachtii* populations were sampled between 2014 and 2016 in a single field in seven different countries and localities representing contrasted climatic zone : Morocco (Mor; Al Aroui), Spain (Spa; Lebridja), France (Fra; Prunay), Germany (Ger; Schlanstedt), Austria (Aus; Tadten), Poland (Pol; Radlowek), and Ukraine (Ukr; Teofipol). Each population corresponded to a single soil sample constituted of a minimum amount of 2 kg and a minimum of 10 elementary samples collected randomly in the field between 0 and 20 centimeters deep. The cysts extracted from each of these seven soil samples were multiplied one time in standardized conditions, (20°C, room chamber, 16:8) for two to four generations on the cultivar Ardan. Newly formed cysts were then stored at 5°C in moistened sandy soil.

### Genetic Diversity

The seven *H. schachtii* populations were genotyped using eight microsatellite markers developed by Montarry et al. (2015): Hs33, Hs36, Hs55, Hs56, Hs68, Hs84, Hs111, and Hs114. Between 21 and 39 individuals per population were successfully genotyped using only one larva (i.e., 1 s-stage juvenile) per cyst, in order to limit biases due to sibling relationships. DNA of each larva was extracted with a procedure using sodium hydroxide and proteinase K, following Boucher et al. (2013).

Three multiplex panels were defined to genotype the 207 individuals at the eight loci. PCR was performed in a 5 μL volume containing 1X of Type-it Microsatellite PCR kit, 0.4 μM of primer mix and 1 μL of template DNA. Cycling conditions included an initial denaturation at 95°C for 5 min, followed by 30 cycles of denaturation at 95°C for 30 s, annealing at 58°C for 90 s and extension at 72°C for 30 s, followed by a final extension at 60°C for 30 min. PCR products were then diluted to 1:10 in sterile water and 3 μL of this dilution were mixed with 0.05 μL of GeneScan 500 LIZ Size Standard (Applied Biosystems) and 5 μL of formamide (Applied Biosystems). Analyses of PCR products were conducted on a ABI Prism^®^ 3130xl sequencer (Applied Biosystems).

Allele sizes were determined with the automatic calling and binning module of GeneMapper v4.1 (Applied Biosystems), and complemented by a manual examination of irregular results. Samples showing dubious genotypes were tested again.

Genetic diversity in each *H. schachtii* population was estimated using GENETIX 4.05.2 (Belkhir et al., 2004) as the unbiased gene diversity (Hnb) (Nei, 1978). Hnb corresponds to the average probability across loci to draw at random different alleles in the same population.

### Hatching at Different Temperatures

For the seven *H. schachtii* populations, larvae emergence was investigated at three temperatures: 11, 17, and 23°C, which cover the range of temperatures encounter by European *H. schachtii* populations, from the North to the South, during the cropping period. For each population and each temperature, six independent replicates were performed. Each replicate consisted of a pool of four cysts, calibrated in size between 450 and 500 μm. Cysts were placed on a sieve of 250 μm, allowing the active passage of larvae. Each sieve was put in one well of a 24-well plate containing root exudates of sugar beet (cv. Ardan). Plates were placed in the dark in three different climate chambers set at the respective temperatures. Temperature within each climate chamber was recorded every hour using thermo-tracers (ThermoTracer, Oceasoft, Montpellier, France – Supplementary Figure S1). The number of hatched larvae was counted after 1, 2, 3, 4, 7, 11, 15, 19, 23, 28, and 33 days, the exudate being changed at each date. The number of unhatched eggs was assessed at the end of the experiment in order to calculate the percentage of hatching.

### Multiplication at Different Temperatures

For the seven *H. schachtii* populations, *H. schachtii* multiplication was investigated at three temperatures: 11, 17, and 23°C. For each population and each temperature, 27 independent replicates were performed, i.e., 27 sugar beet plants of the cultivar Ardan. Plants were grown in a 4:1 sand-kaolin mixture watered with a nutrient solution (Hakaphos: NPK 15/10/15). Kaolin was used to aerate the sand and thus to promote the mobility of *H. schahctii* larvae. Each plant was inoculated at the two-leaf stage with 1 mL of a suspension of larvae concentrated at 500 larvae.mL^-1^. Plants were placed in three different climate chambers set at the respective temperatures with a photoperiod of 16 h. Temperature within each climate chamber was recorded every hour using thermo-tracers (ThermoTracer, Oceasoft, Montpellier, France – Supplementary Figure S1). Plants were cut at the end of the cycle, when white females were well formed and contained eggs, i.e., after 4 weeks at 23°C, after 5 weeks at 17°C, and after 9 weeks at 11°C. Plants were kept several weeks in their climatic chamber until the maturation of cysts, i.e., three, four and six additional weeks at 23, 17, and 11°C, respectively. Newly formed cysts were extracted by filtering the soil through two sieves (800 and 250 μm) and counted under a stereomicroscope.

### Hatching Following Storage at Different Temperatures

Each *H. schachtii* population was multiplied one generation on the sugar beet cultivar Ardan and newly formed cysts were then stored during 2 months at four temperatures: -3, 4, 10, and 25°C, which cover the range of temperatures encounter by European *H. schachtii* populations, from the North to the South, during the inter-cropping period. For each population and each temperature, six independent replicates were performed. Each replicate consisted of a pool of four cysts, calibrated for their size between 450 and 500 μm, and placed in boxes containing 33 g of sand and 7 mL of water. Boxes were stored in the dark in incubators set at the respective temperatures. Temperature within each incubator was recorded every hour using thermo-tracers (ThermoTracer, Oceasoft, Montpellier, France – Supplementary Figure S1). After the storage period, each pool of four cysts was placed on a sieve of 250 μm, allowing the active passage of larvae, and each sieve was put in a 24-well plate containing root exudates of sugar beet (cv. Ardan). Plates were placed in the dark at 20°C (± 1) in an incubator. The number of hatched larvae was counted after 1, 2, 3, 4, 7, 11, 15, 19, 23, and 28 days, the exudate being changed at each date. The number of unhatched eggs was assessed at the end of the experiment in order to calculate the percentage of hatching.

### Statistical Analysis

In order to take into account the hatching dynamic in its entirety, and not only the final point of the hatching curve, the Area Under the Hatching Curve (AUHC) was calculated for each replicate. For each population, and for each measured traits (i.e., AUHC, number of produced cysts and AUHC after storage), the temperature effect was tested through a one-way ANOVA and multiple comparisons of means were done with the Tukey contrasts test (α = 0.05). Normality and homogeneity of variances were checked using the Shapiro–Wilk and the Leven tests, respectively.

Standardized niche breadth (*B_A_*) – a parameter estimating the level of ecological specialization of a species (or a population) – was calculated for each *H. schachtii* population using the standardized index developed by Levins (1968):

BA=1n−1[1∑pi2−1]

where *p_i_* is the proportion of the measured fitness trait into the environment *i* and *n* the total number of tested environments. In this study, *n* = 3 for the hatching and the multiplication at different temperatures (11, 17, and 23°C) and *n* = 4 four for the hatching following storage at different temperatures (-3, 4, 10, and 25°C). The standardized niche breadth varies from 0 to 1. A low niche breadth points to a high level of specialization (i.e., a specialist species) and an high niche breadth to a low level of specialization (i.e., a generalist species).

The relationships between the level of specialization (*B_A_*) and the genetic diversity (Hnb) were tested with the Pearson’s product-moment correlation. All statistical analyses were performed using the R software version 3.3.3 (R Development Core Team, 2017).

## Results

### Hatching and Multiplication at Different Temperatures

**FIGURE 1:**
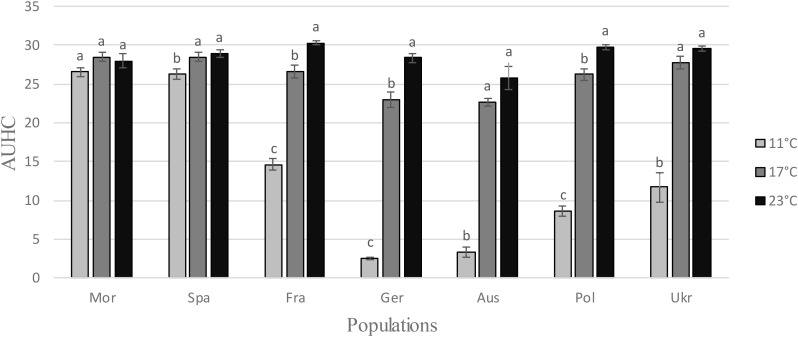
Area Under the Hatching Curve (AUHC) at different temperatures (11, 17, and 23°C) for each *Heterodera schachtii* population (mean values ± standard deviations). Letters represent the homogenous groups identified by the Tukey contrasts test at the 5% threshold.

ANOVAs revealed a very strong temperature effect for five *H. schachtii* populations, Fra, Ger, Aus, Pol, and Ukr (Table 1 and Figure 1), showing that the hatching pattern was very different at 11°C: larvae emergence was lower at 11°C than at 17 or 23°C, in terms of AUHC (Figure 1) but also in terms of cumulative hatching at the end of the experiment (Supplementary Figure S2). Conversely, the temperature effect was very small or even non-significant for the two remaining populations, Mor and Spa (Table 1 and Figure 1), for which larvae emergence (i.e., AUHC – Figure 1 – and cumulative hatching at the end of the experiment – Supplementary Figure S2) was similar whatever the temperature.

**Table 1:** Results from the analyses of variance (ANOVAs) assessing the temperature effect on the three explored life-history traits (AUHC at different temperatures, on the number of cysts produced at different temperatures and on AUHC after storage at different temperatures) for each *Heterodera schachtii* populations.

Population	Source of variation	AUHC			Number of cysts			AUHC after storage		
		df	*F*-value	*P* > *F*	df	*F*-value	*P* > *F*	df	*F*-value	*P* > *F*
**Mor**	Temperature effect	2	2.00	0.1700	2	12.67	<0.0001^∗∗∗^	3	20.31	<0.0001^∗∗∗^
	Error	15			74			20		
**Spa**	Temperature effect	2	10.70	0.0013^∗∗^	2	4.33	0.0166^∗^	3	12.10	<0.0001^∗∗∗^
	Error	15			75			20		
**Fra**	Temperature effect	2	156.60	<0.0001^∗∗∗^	2	0.44	0.6480	3	11.84	0.0001^∗∗∗^
	Error	15			74			20		
**Ger**	Temperature effect	2	407.30	<0.0001^∗∗∗^	2	11.15	<0.0001^∗∗∗^	3	0.99	0.4170
	Error	15			74			20		
**Aus**	Temperature effect	2	163.50	<0.0001^∗∗∗^	2	4.28	0.0174^∗^	3	4.69	0.0122^∗^
	Error	15			76			20		
**Pol**	Temperature effect	2	327.60	<0.0001^∗∗∗^	2	22.49	<0.0001^∗∗∗^	3	15.64	<0.0001^∗∗∗^
	Error	15			74			20		
**Ukr**	Temperature effect	2	69.29	<0.0001^∗∗∗^	2	11.16	<0.0001^∗∗∗^	3	9.00	0.0006^∗∗∗^
	Error	15			75			20		

*The statistically significant effects are indicated as ^∗^*P* < 0.05, ^∗∗^*P* < 0.01, and ^∗∗∗^*P* < 0.001*.

**FIGURE 2:**
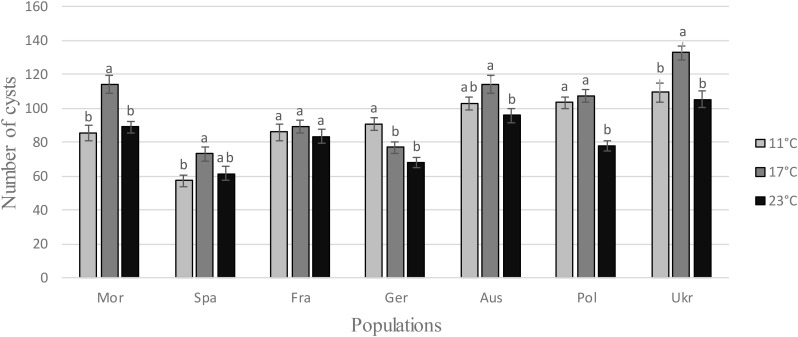
Number of cysts obtained from a multiplication at different temperatures (11, 17, and 23°C) for each *H. schachtii* population (mean values ± standard deviations). Letters represent the homogenous groups identified by the Tukey contrasts test at the 5% threshold.

Regarding the number cysts produced on sugar beet plants at different temperatures, ANOVAs revealed a temperature effect for all populations, except for the French one, showing that *H. schachtii* populations produced more cysts at the intermediary temperature, i.e., 17°C (Table 1 and Figure 2).

### Hatching Following Storage at Different Temperatures

**FIGURE 3:**
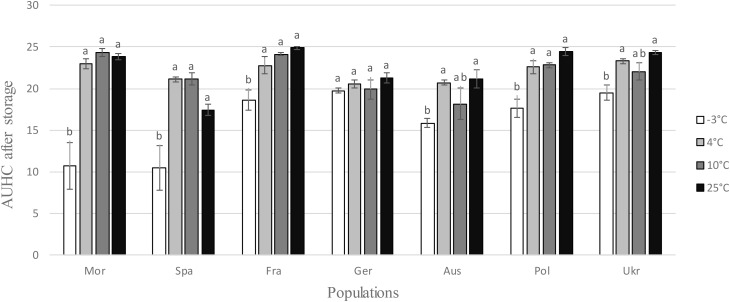
Area Under the Hatching Curve (AUHC) after storage at different temperatures (–3, 4, 10, and 25°C) for each *H. schachtii* population (mean values ± standard deviations). Letters represent the homogenous groups identified by the Tukey contrasts test at the 5% threshold.

After a prior exposure for 2 months to different storage temperatures, the hatching experiment was performed at 20°C (±1) because at this temperature larvae of all populations hatched very well, as showed by the previous experiment (Figure 1). Larvae emergence was reduced after storage at -3°C for all populations except the German one (Table 1 and Figure 3). The difference between the storage step at -3°C and the three other temperatures (4, 10, and 25°C) was much higher for the two Mediterranean populations (Mor and Spa) than for the five others (Fra, Ger, Aus, Pol, and Ukr), both for the AUHC (Figure 3) or the cumulative hatching at the end of the experiment (Supplementary Figure S3).

### Relationship Between Genetic Diversity and Ecological Specialization

**FIGURE 4:**
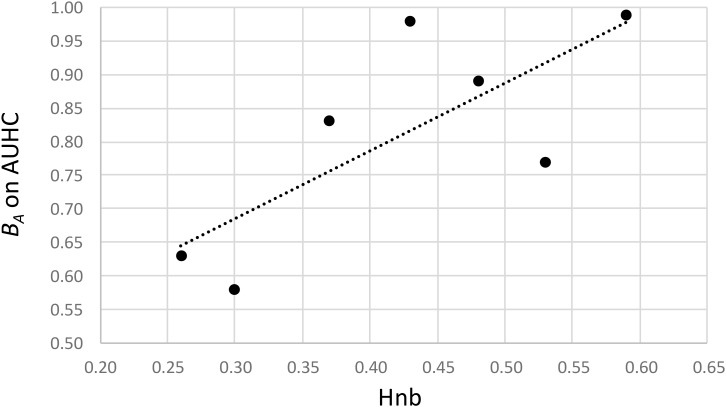
Relationship between the genetic diversity (Hnb) and the standardized niche breadth on the AUHC at different temperatures (*B_A_* on AUHC) calculated of each *H. schachtii* population.

Regarding the hatching (AUHC) at different temperatures, the standardized niche breadth *B_A_* – a parameter to measure how specialized a population is within a given environment – highlighted the generalist status of *H. schachtii* populations from Morocco and Spain (wide niche breath, 0.98 < *B_A_* < 0.99) and showed a lower niche breadth for the five other populations (0.58 < *B_A_* < 0.89), when taking temperatures as different environments (Table 2). Moreover, there was a significant correlation between the genetic diversity and the level of specialization (cor = 0.758; *P* = 0.048), showing that the less diverse populations were more specialized than the more diverse ones, and *vice versa* (Figure 4).

**Table 2:** Genetic diversity (Hnb) and standardized niche breadth (*B_A_*) calculated on AUHC at different temperatures, on the number of cysts produced at different temperatures and on AUHC after storage at different temperatures for each *H. schachtii* populations (Pop).

				B_A_ on the	B_A_ on AUHC
Pop	*n*	Hnb	B_A_ on AUHC	number of cysts	after storage
Mor	38	0.43	0.98	0.97	0.90
Spa	21	0.59	0.99	0.98	0.92
Fra	22	0.48	0.89	0.99	0.98
Ger	37	0.30	0.58	0.98	0.99
Aus	39	0.26	0.63	0.99	0.98
Pol	22	0.53	0.77	0.97	0.98
Ukr	28	0.37	0.83	0.98	0.99

*n indicates the number of genotyped individuals*.

Regarding the multiplication (number of cysts) at different temperatures and the hatching (AUHC) after storage at different temperatures, the standardized niche breadth *B_A_* highlighted the generalist status of all *H. schachtii* populations (0.97 < *B_A_* < 0.99 for the number of cysts and 0.92 < *B_A_* < 0.99 for the AUHC, Table 2) and no correlation with the genetic diversity (cor = -0.373; *P* = 0.410 for the number of cysts and cor = -0.464; *P* = 0.294 for the AUHC).

## Discussion

Although the hatching and development of the beet cyst nematode *H. schachtii* have been already studied (e.g., Griffin, 1988), far less is known about the survival abilities of this species under different climate conditions that can be encountered during the inter-cropping season. The main objectives of the present work were thus to test the effect of temperature and to compare the level of specialization of different *H. schachtii* populations for key traits involved in survival and reproduction: hatching, development in the host plant and hatching after a survival period.

Low temperatures strongly reduced both the hatching pattern and the cumulative hatching rate of five populations (France, Germany, Austria, Poland, and Ukraine): at 11°C, hatching was slowly and was incomplete. Previous studies already showed the same behavior in populations from the same geographical areas (e.g., Vandenbossche et al., 2015). Conversely, temperature had only a small effect on the two southern populations, from Spain and Morocco. This was a surprising result: one could imagine that northern populations should be less constrained by lower temperatures than southern ones, which faced much higher temperature levels over the year. In an unexpected way, temperature appeared thus to act differently according to the geographical origin of populations in the sequence leading to the hatch of larvae. Perry and Gaur (1996) showed that differences related to the host, for instance the age of the host plant, on which the nematodes multiplied could strongly affect their subsequent hatching rate. In our study, cysts all came from a single multiplication cycle on the same sugar beet cultivar, and experiments were performed in controlled conditions. The differences observed in hatching behavior could thus not be assigned to the conditions for obtaining cysts used in this study, but would rather be a clear answer of each population to the thermal conditions applied in the experiment. Indeed, cyst nematode species are all obligate parasites, and their reproductive success mainly depends on the synchronization between hatching and host presence. Consequently, it seems that natural selection has optimized the hatching response to enhance fitness in a different way for northern and southern populations (Spain and Morocco). For these last populations, the most important factor driving hatching may rather be the occurrence of favorable conditions (moisture and presence of root exudates), rather than temperature only.

Regarding the development in the host plant, thermal conditions did not affect each population differentially. In all cases, the number of newly formed cysts matched the usual shape of a thermal performance curve (Schulte et al., 2011), and all populations exhibited higher performances at 17°C. This is consistent with what was already described in the literature for field populations (e.g., Griffin, 1988) and for wild populations of *H. schachtii* (Gracianne et al., 2014). This optimum corresponds also to the optimal temperature for sugar beet cropping. Indeed, even if sugar beet is grown throughout Europe, cropping is always realized under conditions that ensure optimal development and yields. These conditions correspond to the period from April to September in the majority of European countries, but from November to May in Morocco and in the south of Spain.

Most studies only considered the effect of temperature on the multiplication of plant parasites during the epidemic phase, i.e., during the interaction with the host. Here, we also investigated its impact on the survival of the progeny inside the cysts, because the ability to survive when host plants were absent is also an important fitness component submitted to selection (Roff, 1992). In our case study, temperature during the inter-epidemic phases are much more variable than those encountered during the cropping period: they correspond either to very hot summer conditions for the southern populations and to cold winter conditions for the populations of northern and central Europe. In both cases, these periods are characterized by unfavorable conditions for the growth of host plants. We thus exposed cysts of each *H. schachtii* population during 2 months to temperatures from -3 to 25°C, which correspond to the range encountered in natural conditions. The results confirmed the existence of two distinct groups of populations: one (Morocco, Spain) for which survival at a low temperature (-3°C) reduced strongly the subsequent hatching rate, and a second one (all others populations) where survival temperatures had only a limited impact on subsequent hatching. At this point we could therefore conclude, rightly as these populations never encountered such low temperatures, that both southern populations were thus clearly maladapted to survival under cold conditions, while northern populations seemed adapted to survive over a larger range of temperatures, including cold ones.

However, closer examination of the mean standard deviations leads to a more nuanced conclusion. Indeed, southern populations both exhibited greater variation for this trait than northern ones (Figure 3 and Supplementary Figure S3) and seemed to contain some individuals that could survive cold conditions and others that could not (see Supplementary Figure S4 showing the hatching curves for each independent replicate). This observation makes sense in an evolutionary context, where northern populations can be seen as the result of a gradual northward (re)colonization process of Europe after the Last Glacial Maximum (LGM), with the selection among southern individuals of those able to survive the coldest temperatures. Several studies make this hypothesis relevant. First, the south of the Iberian Peninsula is well known as an historical refuge, climatically stable during geological times (Rodrıguez-Sanchez et al., 2008) and therefore during the LGM which corresponded to the latest coldest conditions encountered in Europe. Second, two recent studies tend to prove that wild populations of *H. schachtii* and of its wild hosts *Beta maritima* recolonized the north of Europe following a northward process of migration from the south of the Iberian Peninsula (Leys et al., 2014; Gracianne, 2015; respectively). After the LGM, the following huge increase of temperature pushes northward favorable conditions to the development of host plants and nematodes and make possible the recolonization of northern Europe. While our study was performed with field populations that share only part of their evolutionary history with wild ones, our results matched this scenario rather well. Indeed, we also observed a northward decrease of genetic diversity in our field populations, as expected in the case of sequential founder events associated to recolonization processes and limited gene flow among population (Austerlitz et al., 1997).

As a last challenge, we explored the link between genetic diversity in populations and their ecological specialization, testing the hypothesis that greater genetic diversity is associated with less ecological specialization. Such a relationship was only demonstrated so far between species: in 11 of 14 studies identified by Li et al. (2014), the specialist species showed lower genetic diversity than related generalists. Here, a similar trend can be observed between populations: Spanish and Morrocan populations were both generalists, with a high niche breath and a higher genetic diversity, while Northern populations appeared to be more specialized and less diverse. The generalist character fitted the independence of the hatching and development according to thermal conditions. The association of this generalist trait with higher genetic diversity fitted also well with Gracianne’s scenario, which assumes that Spain and more southern regions kept favorable climate conditions during the LGM, promoting the maintenance of a huge genetic diversity in these areas. The less diversity and the higher specialization to cold climate conditions we observed in northern populations fitted again well with this scenario. The northward recolonization of Europe was associated with the selection for a better capability to survive and a less susceptibility to hatch at low temperatures, maximizing the synchronization between hatching and host presence.

Further investigations are now needed, using wild *H. schachtii* populations, to determine whether the counter-selection of the capability to hatch at low temperatures and the selection of the capability to survive at low temperatures are the result of a fast evolution process in the cultivated compartment (i.e., an adaptation to crop practices in northern Europe), or of a longer process in the wild, associated to the northward recolonization of Europe. Anyway, the high tolerance to contrasted survival conditions observed in northern Europe field populations explains well the presence of *H. schachtii* all around the world where sugar beet is grown (Subbotin et al., 2010). Furthermore, in the framework of global climate change, our data imply that northern populations of *H. schachtii* should not be strongly affected for both survival and development by global warming.

## Data Availability

All data used in this article are available at data.inra.fr (doi: 10.15454/ZKGTAH).

## Author Contributions

LP, CaP, and ChP performed the experiments according to a protocol elaborated jointly by SF, EG, and JM. SF and JM analyzed the data. SF, EG, and JM wrote the text and prepared the figures. All authors edited the paper and have approved the current version.

## Conflict of Interest Statement

The authors declare that the research was conducted in the absence of any commercial or financial relationships that could be construed as a potential conflict of interest.
