# Supplementary material for: Populations of the Beet Cyst Nematode Heterodera schachtii Exhibit Strong Differences in Their Life-History Traits Across Changing Thermal Conditions
Source: Front Microbiol. 2018 Nov 16;9:2801. doi: 10.3389/fmicb.2018.02801 (PMC6250974; doi:10.3389/fmicb.2018.02801)
Supplement: Supplementary file 1 [file Data_Sheet_1.docx]

**Supporting Information, Figure S1** Recorded temperatures (**A**) in the three climatic chambers set at 11°C, 17°C and 23°C with a photoperiod of 16h and (**B**) in the four incubators set at -3°C, 4°C, 10°C and 25°C in the dark.

**Supporting Information, Figure S2** Percentage of hatching (± standard error) measured during 33 days post-inoculation (dpi) at different temperatures (11°C, 17°C and 23°C) for each *H. schachtii* population (Mor, Spa, Fra, Ger, Aus, Pol and Ukr).

**Supporting Information, Figure S3** Percentage of hatching (± standard error) measured during 28 days post-inoculation (dpi) after storage at different temperatures (-3°C, 4°C, 10°C and 25°C) for each *H. schachtii* population (Mor, Spa, Fra, Ger, Aus, Pol and Ukr).

**Supporting Information, Figure S4** Percentage of hatching measured during 28 days post-inoculation (dpi) after storage at -3°C for each of the six independent replicates of the *H. schachtii* populations from Morocco (Mor) and Spain (Spa).
